# Healthy diet attenuates the cardiovascular risk associated with elevated monocytes: a prospective cohort study

**DOI:** 10.3389/fnut.2026.1813578

**Published:** 2026-05-26

**Authors:** Xueguang Lin, Shishuai Xie, Qi Sun, Ying Deng, Jingdong Tang, Hui Zhang, Bo Yu, Shuai Jiang

**Affiliations:** 1 Department of Vascular Surgery, Shanghai Pudong Hospital, Fudan University Pudong Medical Center, Shanghai, China; 2 Shanghai Key Laboratory of Vascular Lesions Regulation and Remodeling, Shanghai, China; 3 Department of Endocrinology and Metabolism, Affiliated Hospital of Nantong University, Nantong, China; 4 State Key Laboratory of Genetic Engineering, Collaborative Innovation Center for Genetics and Development, School of Life Sciences and Human Phenome Institute, Fudan University, Shanghai, China; 5 Department of Vascular Surgery Division, General Surgery Department, Huashan Hospital Affiliated to Fudan University, Shanghai, China

**Keywords:** cardiovascular disease, dietary quality, inflammation, monocytes, prospective cohort study, UK Biobank

## Abstract

**Background:**

Elevated monocyte counts predict cardiovascular disease (CVD) risk, but whether this inflammatory risk is modifiable by dietary patterns remains unknown. We investigated whether dietary quality modifies the association between peripheral monocyte counts and incident CVD and explored potential proteomic mechanisms.

**Methods:**

This prospective cohort study included 26,585 participants from the UK Biobank without baseline CVD. Dietary quality was assessed using a Diet Quality Index (DQI) incorporating processed meat, fish, and plant-based food consumption. Monocyte counts were measured at baseline. Cox proportional hazards models with restricted cubic splines were used to examine the dose–response relationship between monocyte counts and CVD risk, stratified by dietary quality. Joint analyses evaluated combined effects of high monocyte counts and unhealthy diet on CVD outcomes. Proteome-wide interaction analysis was performed to identify plasma proteins linking diet-immune interactions to CVD risk.

**Results:**

During a median follow-up of 16.2 years, 1,834 CVD events occurred. Elevated monocyte counts were associated with increased CVD risk (HR per SD: 1.28, 95% CI: 1.11–1.48, *p* = 0.0009). A significant interaction was observed between monocyte count and dietary quality (*P* interaction = 0.04). Joint analysis revealed that participants with high monocyte counts and unhealthy diet faced the highest risk (HR: 1.18, 95% CI: 1.03–1.35), while those with high monocyte counts but healthy diet showed no excess risk (HR: 1.04, 95% CI: 0.91–1.20). In an exploratory proteome-wide interaction screen, Regenerating islet-derived protein 4 (REG4) was identified as the top nominal interaction signal (P interaction = 2.69 × 10^−5^; FDR q = 0.079), though this did not survive multiple testing correction. High diet quality substantially attenuated the positive association between monocytes and pro-inflammatory REG4 levels observed in unhealthy eaters.

**Conclusion:**

Dietary quality modulates the association between peripheral monocyte counts and cardiovascular disease risk, potentially involving downstream inflammatory proteins such as REG4, though this proteomic finding remains exploratory. For individuals with elevated inflammatory burden, adherence to a healthy diet represents a practical strategy to mitigate excess cardiovascular risk.

## Introduction

Cardiovascular disease (CVD) remains the leading cause of mortality worldwide (1). Despite significant advances in lipid-lowering therapies, a substantial residual cardiovascular risk persists, particularly among individuals with elevated inflammatory burden (2). Accumulating evidence has firmly established inflammation as a critical driver of atherosclerosis, prompting a paradigm shift from viewing CVD solely as a lipid storage disorder to recognizing it as a chronic inflammatory condition (3, 4).

Monocytes, as key effectors of innate immunity, play a pivotal role in this pathogenesis. These circulating cells serve as the primary source of macrophages that populate atherosclerotic plaques, orchestrating inflammatory responses and contributing to plaque destabilization (5, 6). Epidemiological studies consistently demonstrate that elevated peripheral monocyte counts predict incident CVD events independent of traditional risk factors (7, 8). While the recruitment mechanisms—such as the MCP-1/CCR2 axis—are well-characterized, an emerging question is whether this monocyte-driven risk is modifiable by lifestyle interventions (9).

While the deleterious effects of elevated monocytes on cardiovascular health are well-established, an emerging question is whether this inflammatory risk is modifiable by lifestyle interventions, particularly dietary modification. Poor diet quality has been identified as one of the most potent modifiable risk factors for CVD, with dietary patterns accounting for a significant proportion of cardiovascular mortality (10, 11). Evidence-based dietary recommendations emphasize increased consumption of fruits, vegetables, whole grains, nuts, and legumes, while limiting intake of processed meats, refined carbohydrates, saturated fats, and sodium (12). Among various dietary patterns, the Mediterranean diet and Dietary Approaches to Stop Hypertension (DASH) diet have demonstrated consistent cardiovascular benefits in both primary and secondary prevention settings (13, 14).

Recent mechanistic studies have begun to elucidate the complex interplay between diet and immune function, revealing that dietary composition can profoundly influence monocyte biology. Groundbreaking work by Jordan et al. demonstrated that short-term fasting reduces the circulating monocyte pool by modulating hepatic AMPK-PPARα signaling and suppressing systemic CCL2 production (15). Conversely, Western dietary patterns characterized by high saturated fat and simple sugar content promote monocyte polarization toward a pro-inflammatory phenotype, increase sympathetic nervous system activity, and enhance expression of genes involved in inflammatory pathways. These findings suggest that diet may not merely act as a passive risk factor but could actively modulate the relationship between peripheral immunity and cardiovascular outcomes. However, the specific circulating proteins that bridge gut mucosal status with systemic inflammation and atherosclerotic risk remain to be fully identified, warranting investigation into novel, non-heritable molecular pathways.

Despite these insights, a critical knowledge gap remains: it is unknown whether high-quality dietary patterns can attenuate the association between elevated monocyte counts and CVD risk in free-living populations. We hypothesized that adherence to a healthy diet would modify the dose–response relationship between circulating monocytes and cardiovascular outcomes. Using a large prospective cohort, we examined whether high-quality diets could mitigate the excess risk associated with high monocyte burden, providing novel evidence for a targeted strategy to manage immune-mediated cardiovascular risk.

## Methods

### Study population and design

This prospective cohort study utilized data from the UK Biobank, a large-scale population-based biomedical database containing comprehensive health information from approximately 502,000 participants aged 40–69 years recruited between 2006 and 2010 across the United Kingdom (16). The UK Biobank received ethical approval from the North West Multi-Centre Research Ethics Committee (reference: 11/NW/0382), and all participants provided written informed consent. This study was conducted in accordance with the principles of the Declaration of Helsinki.

The derivation of the analytic sample is summarized in Supplementary Table 11. Briefly, from 502,356 participants in the initial dataset, we sequentially restricted the sample to those with complete dietary variables, plausible energy intake, and complete baseline covariates. We then applied complete-case requirements for follow-up, outcome ascertainment, monocyte count, and key covariates, yielding a final analytic sample of 26,585 participants for the monocyte-CVD analysis. A nested proteomic subsample of 2,738 participants with available Olink measurements was used for the exploratory proteomic analyses.

### Dietary quality assessment

Dietary intake was assessed at baseline using a web-based 24-h dietary recall questionnaire (Oxford WebQ), which has been validated against interviewer-administered recalls and demonstrates good reproducibility (16). We constructed a Diet Quality Index (DQI) based on three dietary components selected *a priori* for their relevance to inflammation and cardiovascular health: processed/red meat intake, fish intake, and plant-based food intake.

The DQI was derived as follows. For processed and red meat, higher intake was assigned lower scores, with 0 servings/week corresponding to 10 points and ≥4 servings/week corresponding to 0 points; intermediate intakes were scored linearly as: score = 10 − (servings/week × 2.5). For fish, the weekly intake of oily and white fish was summed and multiplied by 3, capped at 10 points. For plant-based foods, the weekly intake of legumes and whole grains (including wholemeal bread, oat cereals, and muesli) was summed and multiplied by 2, capped at 10 points. The total DQI was calculated as [(meat score + fish score + plant score) / 30] × 100, yielding a score from 0 to 100, with higher values indicating better dietary quality.

To assess the robustness of DQI construction, we additionally examined alternative DQI formulations in sensitivity analyses, including an equal-weight DQI, an extended DQI incorporating additional diet-related components, and leave-one-out variants excluding one core component at a time. For comparability across versions, each DQI was standardized to a z score before interaction modeling. Detailed component definitions and scoring algorithms are provided in Supplementary Table 5, and the corresponding sensitivity results for alternative DQI formulations are shown in Supplementary Table 4.

For the primary analyses, participants were categorized into high versus low dietary quality groups using the median DQI as the cutpoint. Sensitivity analyses also considered alternative categorizations of the DQI.

### Monocyte count measurement

Peripheral blood samples were collected at baseline assessment centers following standardized protocols. Complete blood counts, including absolute monocyte counts, were measured using automated hematology analyzers (Beckman Coulter LH750) within 24 h of sample collection. Monocyte counts were expressed as cells × 109/L. Quality control procedures included regular calibration, participation in external quality assurance schemes, and exclusion of samples with clotting or hemolysis (17). For analytical purposes, monocyte counts were examined both as a continuous variable (per standard deviation increase) and categorized into quartiles.

### Outcome ascertainment

The primary outcome was incident cardiovascular disease (CVD), defined as the first occurrence during follow-up of a composite cardiovascular event including ischemic heart disease (ICD-10: I20-I25), heart failure (ICD-10: I50), and cerebrovascular disease events (ICD-10: I61, I63, I64, I65, I66, and I672). Outcomes were ascertained through linkage to national health registries, including Hospital Episode Statistics for England and Wales, Scottish Morbidity Records, the Patient Episode Database for Wales, and death registries from the Office for National Statistics and National Records of Scotland. Event dates were defined as the date of first qualifying hospital admission or death, whichever occurred first. Participants were followed from baseline assessment until the first incident CVD event, death from other causes, loss to follow-up, or administrative censoring on 31 December 2022, whichever came first.

### Covariates

Baseline covariates were selected *a priori* based on their established associations with both monocyte counts and cardiovascular outcomes. Demographic variables included age (continuous), sex, and ethnicity (white/non-white). Socioeconomic status was assessed using the Townsend Deprivation Index, a composite measure of material deprivation based on unemployment, non-car ownership, non-home ownership, and household overcrowding.

Lifestyle factors included smoking status (never, previous, current), alcohol consumption frequency (never, occasional [1–3 times/month or special occasions], regular [≥1 time/week]), physical activity (metabolic equivalent task minutes/week), and total energy intake (kJ/day). Anthropometric measurements included body mass index (BMI, kg/m2), measured by trained staff using standardized protocols.

Clinical measurements encompassed systolic and diastolic blood pressure (mean of two automated readings), prevalent hypertension (self-reported physician diagnosis or antihypertensive medication use), and prevalent diabetes (self-reported physician diagnosis, antidiabetic medication use, or HbA1c ≥ 6.5%). Biochemical markers included fasting glucose, total cholesterol, low-density lipoprotein cholesterol (LDL-C), high-density lipoprotein cholesterol (HDL-C), triglycerides, and high-sensitivity C-reactive protein (CRP), all measured using standard clinical chemistry assays. Medication use included lipid-lowering drugs (primarily statins) and antihypertensive agents, ascertained through self-report and verified against prescription records where available.

### Proteomic profiling and screening

Plasma protein levels were measured using the Olink Explore 1536/3072 platform in a subset of participants. To identify potential molecular mediators of the diet-monocyte interaction, we performed a proteome-wide interaction screening. We fitted linear regression models for each protein: Protein\sim Monocyte \times Diet Quality + Covariates. Proteins were ranked by the *p*-value of the interaction term. The Benjamini–Hochberg false discovery rate (FDR) was applied across all 2,924 proteins tested, with a prespecified significance threshold of FDR q < 0.05. Proteins not meeting this threshold were considered exploratory nominal signals only.

### Statistical analysis

Baseline characteristics were summarized as means with standard deviations for approximately normally distributed continuous variables, medians with interquartile ranges for skewed variables, and frequencies with percentages for categorical variables. Comparisons across dietary quality groups were performed using analysis of variance for continuous variables and chi-square tests for categorical variables.

Cox proportional hazards regression models were used to estimate hazard ratios (HRs) and 95% confidence intervals (CIs) for the associations between monocyte count and incident CVD. Monocyte count was analyzed both as a continuous variable and in quartiles. For tests of linear trend across monocyte quartiles, the median monocyte value within each quartile was assigned to all participants in that quartile and entered as a continuous term in the Cox proportional hazards model. The proportional hazards assumption was evaluated using Schoenfeld residuals and log–log plots. Because some evidence of time dependence was observed for monocyte count in simpler models, an additional time-varying effect analysis was performed as a supplementary assessment. We constructed three sequential models with progressive adjustment: Model 1 adjusted for age and sex; Model 2 additionally adjusted for body mass index, smoking status, and alcohol consumption; and Model 3 further adjusted for socioeconomic status. C-reactive protein (CRP) was not included in the primary adjustment set because it may plausibly act as either a confounder or a mediator in the inflammatory pathway linking monocyte count to CVD; its inclusion was therefore examined in a separate sensitivity analysis.

To examine effect modification by dietary quality, multiplicative interaction terms (monocyte count × dietary quality) were added to the fully adjusted models. Statistical significance of interactions was assessed using likelihood ratio tests comparing models with and without the interaction term. Restricted cubic spline analyses with four knots placed at the 5th, 35th, 65th, and 95th percentiles of the monocyte distribution were used to characterize dose–response relationships and assess potential non-linearity, stratified by dietary quality group. Knot placement followed Harrell’s recommendations to balance flexibility and stability (18).

Joint analyses were performed by creating four mutually exclusive categories based on median splits of monocyte count and dietary quality: (1) low monocyte count + high dietary quality (reference); (2) low monocyte count + low dietary quality; (3) high monocyte count + high dietary quality; and (4) high monocyte count + low dietary quality. Kaplan–Meier curves were used to visualize CVD-free survival across these groups, and differences were assessed using the log-rank test.

Several sensitivity analyses were conducted to assess robustness, including exclusion of participants who developed CVD within the first 2 years of follow-up, exclusion of participants with baseline cancer or chronic inflammatory conditions, stratified analyses by age (<60 vs. ≥60 years) and sex, and use of the monocyte-to-lymphocyte ratio as an alternative inflammatory metric. In addition, Fine-Gray competing-risk regression models were fitted with non-CVD death treated as the competing event.

Exploratory metabolomic analyses were conducted in a subset of participants with available plasma metabolite data to investigate potential pathways related to the observed diet-monocyte interaction. Interactions between dietary quality and major metabolite classes were examined using analogous regression models; these analyses were considered exploratory.

All statistical tests were two-sided, and *p* < 0.05 was considered statistically significant. Analyses were performed using R version 4.3.0 (R Foundation for Statistical Computing, Vienna, Austria), including the survival, rms, ggplot2, and dplyr packages.

## Results

### Baseline characteristics

The study cohort comprised 26,585 participants with a mean age of 56.1 years (SD = 8.06), of whom 55.0% were female. Baseline characteristics stratified by dietary quality are presented in Table 1. Participants in the high diet quality group were more likely to be female and older, and had a lower body mass index compared with those in the low diet quality group (female: 57.8% vs. 52.2%; age: 56.4 vs. 55.8 years; BMI: 26.4 vs. 27.3 kg/m^2^; all *p* < 0.001). They also had more favorable cardiovascular risk profiles, including a lower proportion of current smokers (6.8% vs. 9.3%, *p* < 0.001), lower blood pressure (SBP: 139 vs. 140 mmHg; DBP: 81.3 vs. 82.2 mmHg; both *p* < 0.001), and a more favorable lipid and inflammatory profile (lower LDL cholesterol: 3.60 vs. 3.64 mmol/L; higher HDL cholesterol: 1.52 vs. 1.47 mmol/L; lower triglycerides: 1.38 vs. 1.50 mmol/L; lower C-reactive protein: 1.07 vs. 1.28 mg/L; all *p* < 0.001).

**Table 1:** Baseline characteristics of the study participants stratified by diet quality.

Characteristic	Low quality (*N* = 13,293)	High quality (*N* = 13,292)	Total population (*N* = 26,585)	*p*-value
Age, years, mean (SD)	55.8 (8.09)	56.4 (8.01)	56.1 (8.06)	<0.001
Sex				<0.001
Female	6,945 (52.2%)	7,688 (57.8%)	14,633 (55.0%)	
Male	6,348 (47.8%)	5,604 (42.2%)	11,952 (45.0%)	
BMI, kg/m^2^, mean (SD)	27.3 (4.64)	26.4 (4.35)	26.9 (4.52)	<0.001
Townsend Deprivation Index, median [IQR]	−2.21 [3.58]	−2.19 [3.61]	−2.20 [3.60]	0.957
Missing	27 (0.2%)	25 (0.2%)	52 (0.2%)	
Smoking Status				<0.001
Never	7,468 (56.2%)	7,726 (58.1%)	15,194 (57.2%)	
Previous	4,567 (34.4%)	4,645 (34.9%)	9,212 (34.7%)	
Current	1,233 (9.3%)	898 (6.8%)	2,131 (8.0%)	
Missing	25 (0.2%)	23 (0.2%)	48 (0.2%)	
Alcohol Consumption				0.66
Never	687 (5.2%)	740 (5.6%)	1,427 (5.4%)	
Occasional	2,720 (20.5%)	2,746 (20.7%)	5,466 (20.6%)	
Regular	9,881 (74.3%)	9,801 (73.7%)	19,682 (74.0%)	
Missing	5 (0.0%)	5 (0.0%)	10 (0.0%)	
Total Energy Intake, kJ/day, median [IQR]	8,520 [3610]	8,440 [3520]	8,480 [3560]	0.097
Monocyte Count, 10^9^/L, median [IQR]	0.450 [0.200]	0.450 [0.190]	0.450 [0.200]	<0.001
Diet Quality Index (Score), mean (SD)	20.4 (16.2)	65.4 (21.0)	42.9 (29.3)	<0.001
Systolic Blood Pressure, mmHg, mean (SD)	140 (19.3)	139 (19.5)	139 (19.4)	<0.001
Missing	79 (0.6%)	79 (0.6%)	158 (0.6%)	
Diastolic Blood Pressure, mmHg, mean (SD)	82.2 (10.6)	81.3 (10.5)	81.8 (10.5)	<0.001
Missing	79 (0.6%)	79 (0.6%)	158 (0.6%)	
Fasting Glucose, mmol/L, median [IQR]	5.02 [0.644]	5.01 [0.632]	5.02 [0.637]	0.01
Total Cholesterol, mmol/L, mean (SD)	5.81 (1.11)	5.78 (1.09)	5.79 (1.10)	0.149
LDL Cholesterol, mmol/L, mean (SD)	3.64 (0.846)	3.60 (0.831)	3.62 (0.839)	<0.001
HDL Cholesterol, mmol/L, mean (SD)	1.47 (0.378)	1.52 (0.388)	1.50 (0.384)	<0.001
Triglycerides, mmol/L, median [IQR]	1.50 [1.11]	1.38 [0.983]	1.43 [1.04]	<0.001
C-Reactive Protein, mg/L, median [IQR]	1.28 [2.02]	1.07 [1.64]	1.18 [1.83]	<0.001
Incident CVD Events				
No Event	12,306 (92.6%)	12,445 (93.6%)	24,751 (93.1%)	0.003
Incident CVD	987 (7.4%)	847 (6.4%)	1834 (6.9%)	

Values are presented as mean (standard deviation) for continuous variables with normal distribution, median [interquartile range] for skewed variables, and number (percentage) for categorical variables. BMI, body mass index; TDI, Townsend deprivation index; SBP, systolic blood pressure; DBP, diastolic blood pressure; TC, total cholesterol; TG, triglycerides; LDL-C, low-density lipoprotein cholesterol; HDL-C, high-density lipoprotein cholesterol; CRP, C-reactive protein; DQI, diet quality index; CVD, cardiovascular disease. *p*-values were calculated using Student’s t-test or Mann–Whitney U test for continuous variables, and Chi-square test for categorical variables. Missing data for all covariates were <1% and were excluded from the descriptive analysis.

Monocyte count was similar between diet quality groups, with an overall median value of 0.450 × 10^9^/L (IQR = 0.200 × 10^9^/L; *p* < 0.001). Participants in the high diet quality group also had a lower incidence of cardiovascular disease events during follow-up compared with those in the low diet quality group (6.4% vs. 7.4%, *p* = 0.003).

### Association between monocyte count and cardiovascular outcomes

During follow-up, incident cardiovascular events (including myocardial infarction, stroke, and cardiovascular death) were recorded and adjudicated. Cause-specific Cox proportional hazards models demonstrated a significant association between higher monocyte counts and incident CVD (Table 2). When modeled as a continuous variable, monocyte count was associated with incident CVD in the age- and sex-adjusted model (Model 1), with an HR of 1.38 (95% CI: 1.20–1.59, *p* < 0.0001). The association remained statistically significant after further adjustment for body mass index, smoking status, and alcohol consumption (Model 2; HR 1.29, 95% CI: 1.11–1.49, *p* = 0.0007), and after additional adjustment for socioeconomic status (Model 3; HR 1.28, 95% CI: 1.11–1.48, *p* = 0.0009). Results from the CRP-adjusted sensitivity analysis were directionally consistent and are presented in Supplementary Table 8. The proportional hazards assumption was formally evaluated using Schoenfeld residuals. Although the global tests were not statistically significant in the primary adjusted models (Model 3: *p* = 0.097; Supplementary Table 6), the monocyte term showed some evidence of time dependence in simpler models. We therefore additionally examined a time-varying effect model (Supplementary Figure 1), which suggested that the association between monocyte count and incident CVD was stronger during earlier follow-up and attenuated over time. Accordingly, the Cox hazard ratios are interpreted as average effects over follow-up.

**Table 2:** Association between monocyte counts and incident cardiovascular events (CVD) in UKB cohort (cause-specific Cox).

Exposure category	N	Events, n (%)		Model 1 (HR, 95% CI)	*p* value	P for trend	Model 2 (HR, 95% CI)	*p* value	P for trend	Model 3 (HR, 95% CI)	*p* value	P for trend
Monocyte counts	26,585	1834 (6.9%)	Continuous	1.38 (1.20–1.59)	< 0.0001	0.0005	1.29 (1.11–1.49)	0.0007	0.0087	1.28 (1.11–1.48)	0.0009	0.0098
	6,646	296 (4.5%)	Q1	1.00 (Reference)			1.00 (Reference)			1.00 (Reference)		
	6,646	311 (4.7%)	Q2	0.92 (0.53–1.62)			0.88 (0.50–1.54)			0.87 (0.50–1.53)		
	6,646	398 (6.0%)	Q3	1.08 (0.64–1.85)			0.99 (0.58–1.69)			1.00 (0.59–1.70)		
	6,647	829 (12.5%)	Q4	1.94 (1.20–3.13)			1.61 (0.99–2.61)			1.59 (0.98–2.58)		

HR, hazard ratio; CI, confidence interval; Ref, reference. Model 1 was adjusted for age and sex. Model 2 was additionally adjusted for body mass index, smoking status, and alcohol consumption. Model 3 was additionally adjusted for socioeconomic status. P for trend was calculated by assigning the median monocyte value within each quartile to all participants in that quartile and entering this ordered variable as a continuous term in the Cox proportional hazards model. In the cause-specific Cox model, participants who died from causes other than cardiovascular disease were censored at the time of death. The continuous and quartile analyses were conducted in the same analytic sample (*N* = 26,585; incident CVD events = 1,834). A CRP-adjusted sensitivity model, additionally adjusted for log-CRP, is reported in Supplementary Tables 8, 9.

Consistent results were observed when monocyte count was categorized into quartiles. Compared with participants in the lowest quartile (Q1, reference), those in the highest quartile showed elevated risk of incident CVD: Q4 HR 1.59 (95% CI: 0.98–2.58, *p* = 0.0605) in Model 3 (P for trend = 0.0098). Although HRs for Q2/Q3 were near null and Q4 was borderline significant in the fully adjusted model, the trend test remained significant, as trend analysis uses ordered quartile data as a continuous variable rather than independent pairwise comparisons against Q1. The association was thus mainly driven by the highest quartile. The sequential attenuation in effect estimates across models was primarily attributable to confounding adjustment; variance inflation factors for all covariates were below 3, indicating no evidence of problematic multicollinearity (Supplementary Table 7). For transparency, we note that during revision an outcome specification error was identified in the original Table 2 analysis. All Cox proportional hazards models shown in Table 2 were therefore re-run using the corrected outcome definition, and the revised Table 2 reflects these corrected analyses.

### Diet quality modifies the monocyte-CVD association

**Figure 1:**
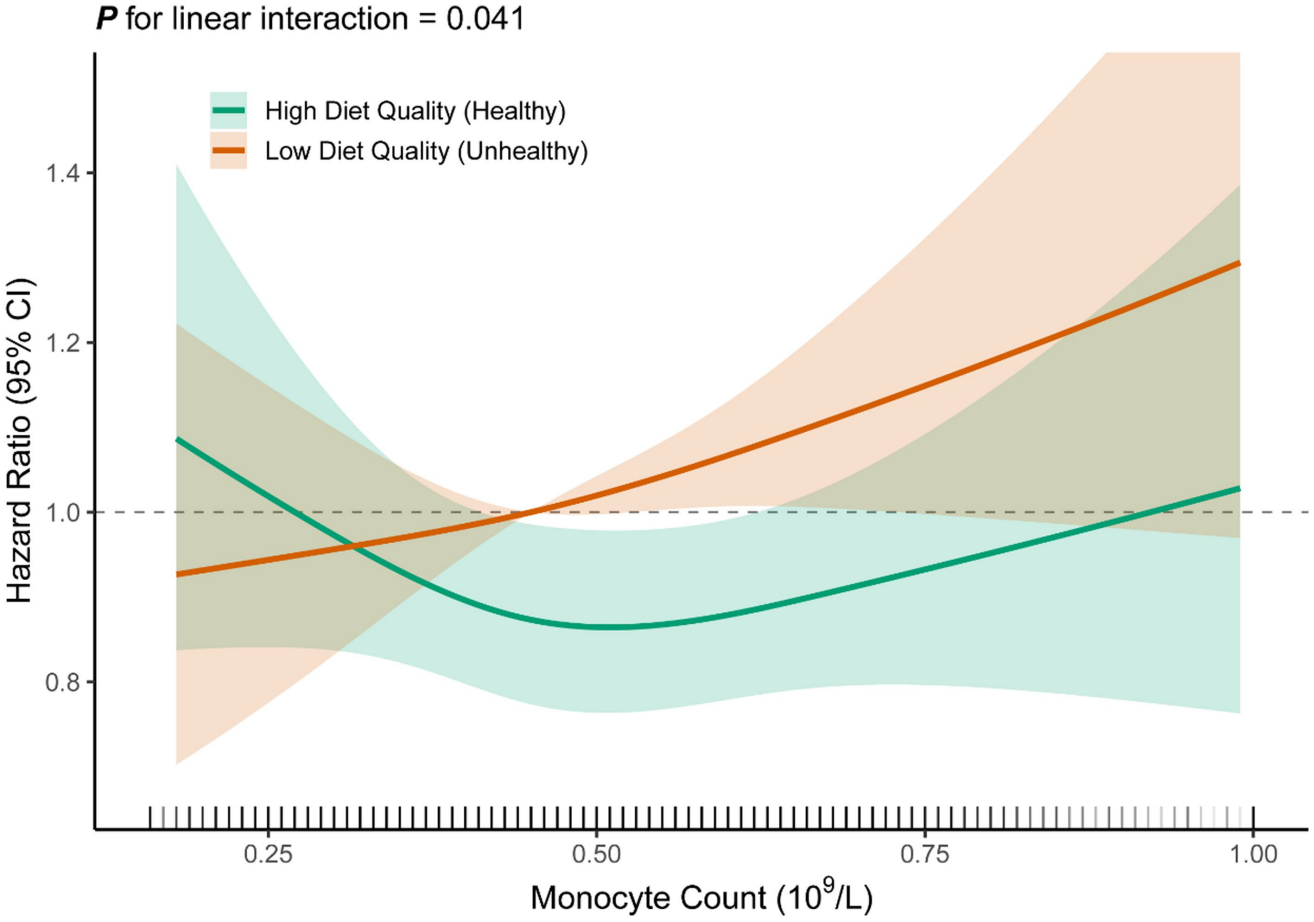
Joint association of monocyte count and diet quality with the risk of incident CVD. Restricted cubic spline (RCS) curves showing the hazard ratios (HRs) for incident CVD according to monocyte count, stratified by diet quality. The red line represents participants with low diet quality (unhealthy diet), and the green line represents participants with high diet quality (healthy diet). Shaded areas indicate 95% confidence intervals. The model was adjusted for age, sex, body mass index, smoking status, alcohol consumption, and socioeconomic status. The reference point (HR = 1.0) was set at the median monocyte count (0.45 × 10^9^/L) for each group to compare the slopes of the risk association. A significant linear interaction was observed between monocyte count and diet quality (*P* for linear interaction = 0.041), indicating that the association between elevated monocyte count and CVD risk was more pronounced in the low diet quality group compared to the high diet quality group.

Diet quality modified the association between monocyte count and incident CVD, though the interaction term was of borderline statistical significance (*P* for linear interaction = 0.041; Figure 1). This finding should be interpreted as suggestive and hypothesis-generating, pending replication in independent cohorts. In restricted cubic spline analyses (reference set at each diet group’s median monocyte count, 0.45 × 10^9^/L), participants with low diet quality showed a steadily increasing CVD risk with rising monocyte counts, with the upward trend becoming more evident at higher monocyte levels. In contrast, among those with high diet quality, the monocyte–CVD association was substantially attenuated: the curve was comparatively flatter across most of the observed monocyte range, indicating smaller changes in CVD risk as monocyte count increased. Overall, these patterns suggest that a healthier diet may buffer the adverse cardiovascular risk associated with elevated monocyte levels. The interaction was also consistent across alternative DQI constructions, including equal-weight, extended, and leave-one-out versions (Supplementary Table 4).

### Joint effects and survival analysis

**Figure 2:**
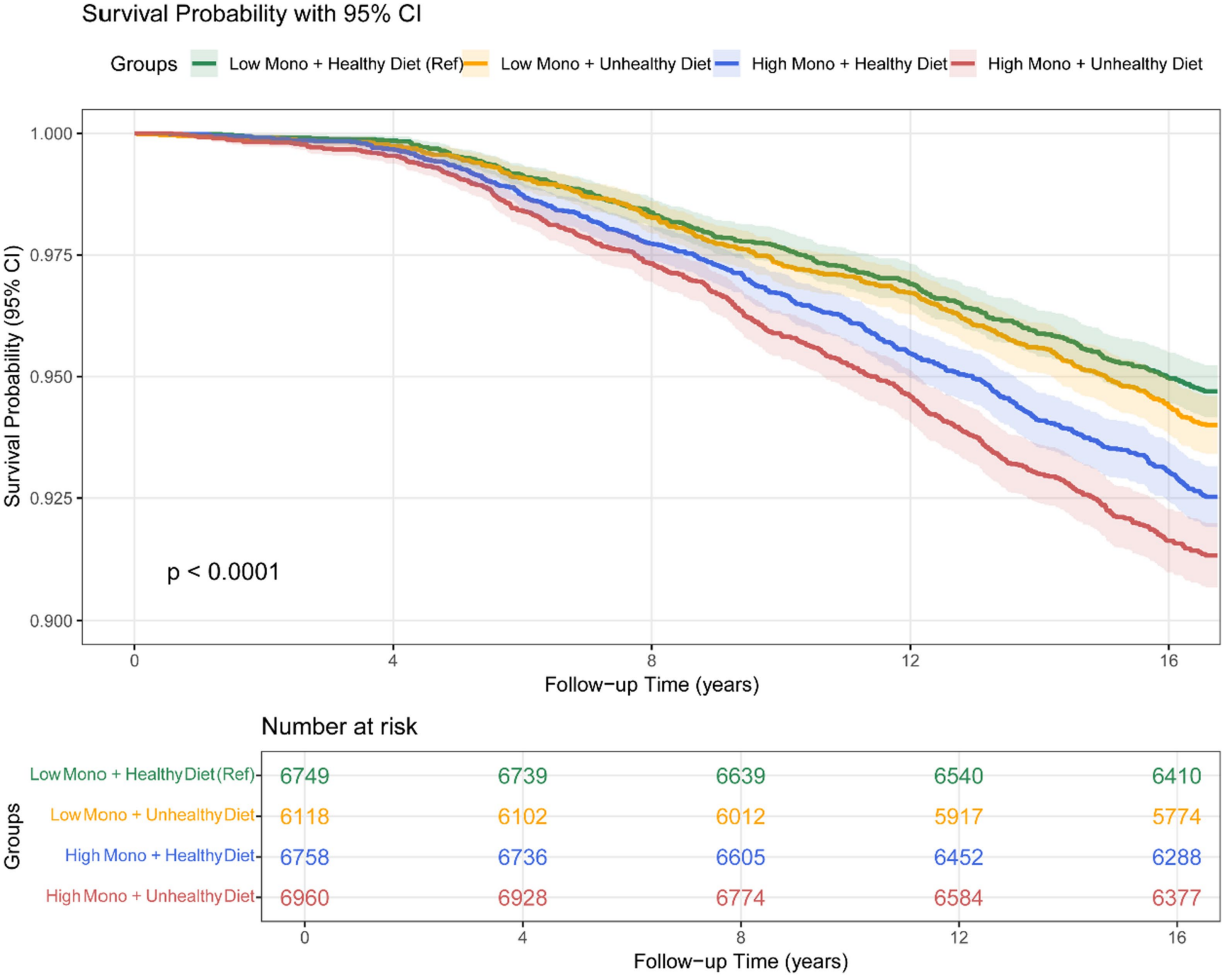
Kaplan–Meier survival estimates for incident cardiovascular disease stratified by the combination of peripheral monocyte counts and diet quality. The curves show the probability of CVD-free survival over a 16-year follow-up period. Participants were categorized into four groups: Low Monocytes + Healthy Diet (Green line, Reference); Low Monocytes + Unhealthy Diet (Yellow line); High Monocytes + Healthy Diet (Blue line); and High Monocytes + Unhealthy Diet (Red line). The shaded areas represent 95% confidence intervals. The table below the graph indicates the number of participants at risk at each time point. The *p*-value (*p* < 0.0001) was derived from the Log-rank test, indicating significant differences in survival distributions among the four groups.

**Figure 3:**
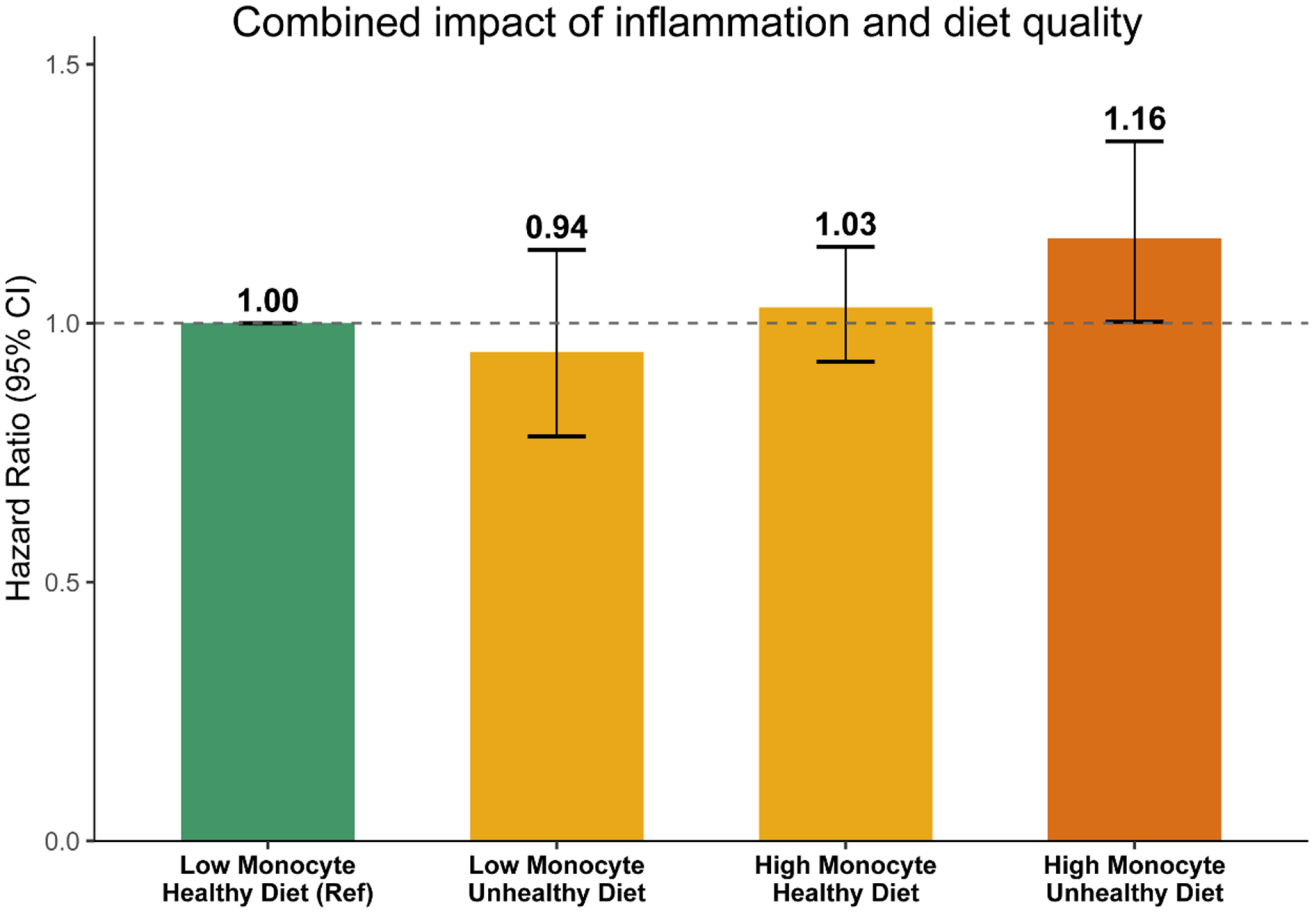
Joint association of monocyte count and diet quality with the risk of incident CVD. Bar chart illustrating the hazard ratios (HRs) and 95% confidence intervals (CIs) for incident CVD across four joint categories of monocyte count and diet quality. Participants were classified into groups based on median monocyte count (Low vs. High) and diet quality (Healthy vs. Unhealthy). The “Low Monocyte + Healthy Diet” group served as the reference category (HR = 1.00). The model was adjusted for age, sex, body mass index, smoking status, alcohol consumption, and socioeconomic status. The highest risk was observed in the “High Monocyte + Unhealthy Diet” group, indicating a potential synergistic adverse effect of systemic inflammation and poor diet quality on cardiovascular health.

Kaplan–Meier survival curves stratified by peripheral monocyte count and dietary quality revealed clear differences in survival patterns across the groups (Figure 2). The group with high monocyte counts and an unhealthy diet had the highest risk of CVD (HR: 1.18, 95% CI: 1.03–1.35), compared to the low monocyte count + healthy diet reference group (Figure 3). Numerical estimates corresponding to the joint exposure categories are provided in Supplementary Table 2. Notably, the cardiovascular risk associated with high monocyte counts was attenuated in participants who adhered to a healthy diet (HR: 1.04, 95% CI: 0.91–1.20), suggesting that dietary quality can may attenuate the cardiovascular risk related to elevated monocyte counts.

### Proteomic identification of potential mechanisms

To elucidate the molecular mechanisms underlying this interaction, we conducted a proteome-wide screening. Among 2,900 + plasma proteins analyzed, Regenerating islet-derived protein 4 (REG4) showed the smallest nominal interaction *p*-value in the screen (*P* interaction = 2.69 × 10^−5^); however, after Benjamini–Hochberg FDR correction across 2,924 proteins, the adjusted q-value was 0.079, which did not meet the prespecified FDR threshold of q < 0.05. REG4 is therefore considered the top exploratory interaction signal rather than a proteome-wide significant finding (Supplementary Table 10). The proteomic analyses were conducted in a subsample of 2,738 participants (10.3% of the main analytic cohort) with available Olink data, and results should be interpreted accordingly.

**Figure 4:**
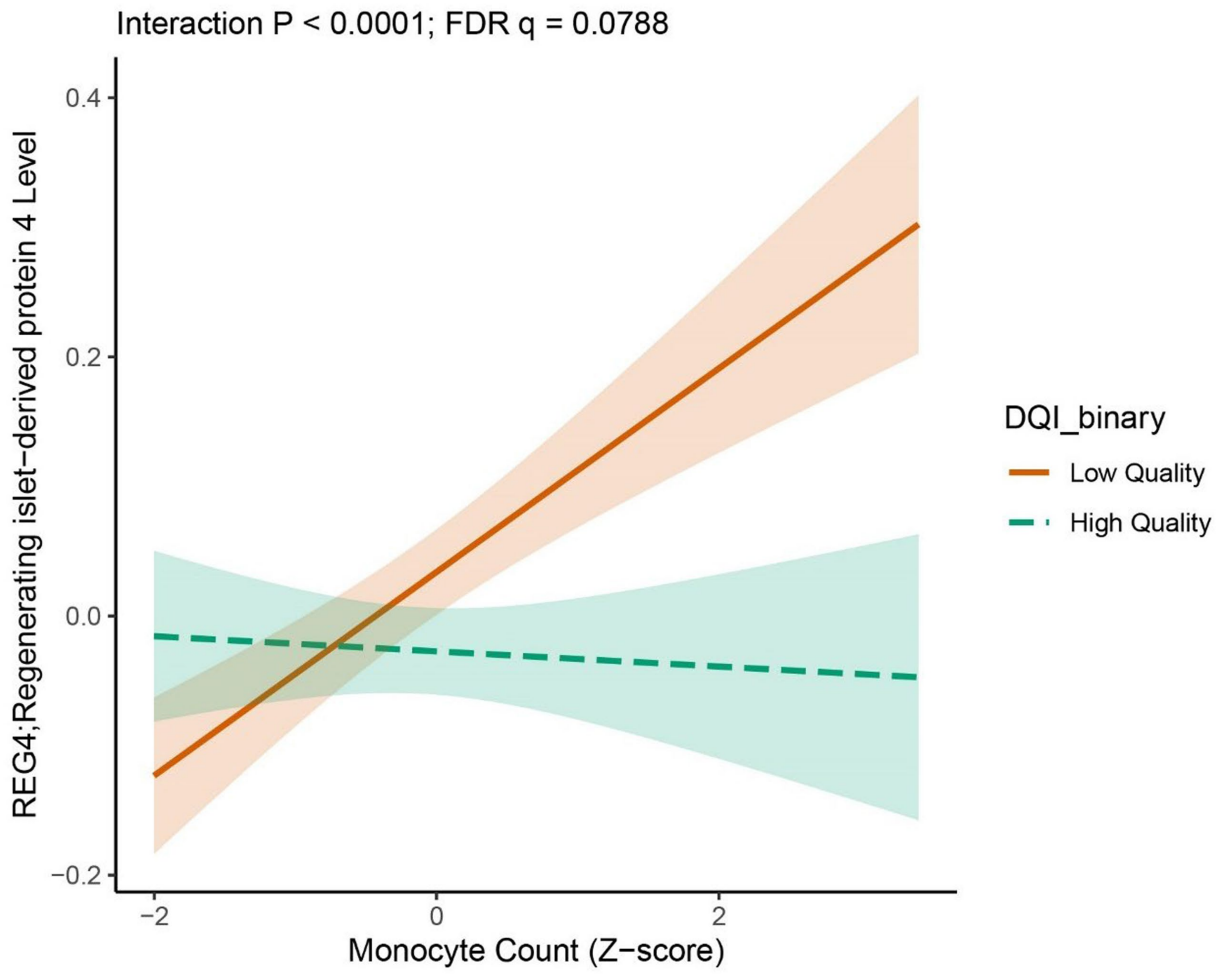
REG4 as a candidate interaction-associated protein. Estimated REG4 protein levels according to monocyte count (Z-score), stratified by binary diet quality (Low Quality vs. High Quality). Note: Lines represent fitted values from the linear regression model; shaded regions show 95% confidence intervals. Model adjusted for age, sex, BMI, smoking status, alcohol consumption, socioeconomic status (SES), and log-transformed C-reactive protein (log-CRP z score). Interaction *p* = 2.69 × 10^−5^; FDR q = 0.0788.

As shown in Figure 4, in participants with low diet quality, elevated monocyte counts were strongly associated with increased plasma REG4 levels (positive slope), suggesting a positive association pattern. Strikingly, this association was effectively blunted in participants adhering to a high-quality diet (flat slope). Other top proteins identified included TIMP2 and INHBB, which are also implicated in tissue remodeling and inflammation.

### Sensitivity analyses

Multiple sensitivity and subgroup analyses supported the robustness of the observed monocyte–diet interaction, including exclusion of early events, subgroup analyses by sex and age (Supplementary Table 1). The diet-monocyte interaction remained significant after excluding participants with baseline CVD (*P* interaction = 0.03), those who developed CVD within the first 2 years of follow-up (*P* interaction = 0.04), and when stratified by sex, age groups, and baseline statin use. Additional adjustment for other white blood cell subtypes and inflammatory markers did not materially alter the association. Exploratory metabolomic analysis did not identify specific plasma metabolites that significantly mediated the diet-monocyte interaction (Supplementary Table 13), suggesting that the protective mechanisms may operate through pathways not captured by conventional plasma metabolite profiling, such as gut microbiome modulation or immune cell epigenetic modifications. Furthermore, Fine–Gray competing-risk analyses treating non-CVD death as a competing event yielded materially similar results to the primary cause-specific Cox models (Supplementary Table 12). Given that both monocyte count and dietary quality were assessed at baseline, the observed associations may represent conservative estimates if within-person variability over time introduced non-differential measurement error.

## Discussion

In this large prospective cohort study, we provide population-based evidence that dietary quality may modify the association between peripheral monocyte counts and incident cardiovascular disease risk. Elevated monocyte counts were associated with higher incident CVD, and this association appeared attenuated among participants with healthier dietary patterns. These findings support diet quality as a potentially important modifier of inflammation-related cardiovascular risk, although replication in independent cohorts is needed.

### Principal findings in context of existing evidence

Our finding that elevated monocyte counts predict CVD aligns with extensive prior evidence documenting the prognostic significance of peripheral inflammatory cell counts (19, 20). Monocytes serve as the primary cellular reservoir for tissue macrophages in atherosclerotic plaques, where they drive lipid accumulation, inflammatory cytokine production, and extracellular matrix degradation (21). Recent mechanistic studies have demonstrated that specific monocyte subsets, particularly the intermediate CD14^+^+CD16^+^ population, exhibit heightened pro-inflammatory characteristics and show the strongest associations with adverse cardiovascular outcomes (22, 23). However, while the pathogenic role of monocytes in atherosclerosis is well-established, whether this risk can be modified through non-pharmacological interventions has remained uncertain.

Our demonstration that dietary quality attenuates monocyte-associated CVD risk extends emerging mechanistic insights into population health. Jordan et al. demonstrated that short-term fasting reduces circulating monocyte pools through hepatic AMPK-PPARα signaling and suppression of systemic CCL2 production (15), while Western dietary patterns promote monocyte polarization toward pro-inflammatory phenotypes (24). Our population-level findings corroborate and extend these experimental observations by demonstrating that habitual dietary patterns exert clinically meaningful effects on the monocyte-CVD relationship over extended follow-up. The dose–response heterogeneity we observed suggests that diet does not merely act as an independent CVD risk factor but fundamentally alters the biological consequences of systemic inflammation.

We also examined the proportional hazards assumption in the sequential Cox models. Although the global Schoenfeld tests were not statistically significant in the primary adjusted models, the monocyte term showed some evidence of time dependence in simpler models. A supplementary time-varying analysis (Supplementary Figure 1) suggested that the association between monocyte count and incident CVD was stronger during earlier follow-up and attenuated over time. The consistency of findings in competing-risk models further supports the robustness of the main results (Supplementary Table 12). Accordingly, the reported Cox hazard ratios should be interpreted as average effects over follow-up rather than strictly constant effects at all time points.

### Potential mechanistic insights: the role of REG4

Our exploratory proteome-wide screen identified REG4 (Regenerating islet-derived protein 4) as the top nominal interaction signal between monocyte count and diet quality, though this did not survive FDR correction. REG4 is primarily characterized in gastrointestinal biology, including colorectal and pancreatic contexts (25), and its relevance to vascular disease remains limited and speculative. One preprint has reported an association between circulating REG4 and incident atherosclerotic cardiovascular disease events (26), but this evidence is preliminary. We therefore present REG4 as a hypothesis-generating candidate warranting further investigation rather than an established mechanistic mediator. No formal mediation analysis was performed, and no causal inference regarding REG4 in the monocyte–CVD pathway can be drawn from the current data.

### Clinical and public health implications

Our findings carry substantial clinical implications for cardiovascular risk stratification and prevention strategies. The concept of “residual inflammatory risk” has gained prominence following the CANTOS trial, which demonstrated that anti-inflammatory therapy targeting interleukin-1β reduces cardiovascular events independent of lipid lowering (27). However, pharmacological anti-inflammatory approaches remain costly, may increase infection risk, and are not universally accessible. Our results suggest that for individuals with elevated monocyte counts—a readily measurable marker of inflammatory burden—dietary intervention represents a practical, accessible, and low-risk strategy to mitigate excess cardiovascular risk.

Importantly, the magnitude of risk attenuation we observed was clinically substantial. The hazard ratio for high monocyte counts in the unhealthy diet group (HR: 1.18) was reduced to non-significance in the healthy diet group (HR: 1.04), representing an approximate 12% reduction in excess risk. This effect size is comparable to many pharmacological interventions and underscores that dietary modification is not merely “good advice” but a critical therapeutic strategy for high-risk individuals. Given the safety profile and broader health benefits of healthy dietary patterns, targeted dietary counseling should be prioritized for patients demonstrating evidence of systemic inflammation.

From a public health perspective, these findings reinforce the importance of population-wide dietary improvement initiatives. The inflammatory component of CVD burden is substantial and may account for a significant proportion of events not prevented by lipid-lowering and antihypertensive therapies (28). Emerging programs such as “food pharmacies” and community-based culinary medicine interventions (29), which increase access to healthy foods and provide practical cooking education, represent promising approaches to translate our findings into practice, particularly in underserved populations where both inflammatory burden and poor dietary quality are prevalent.

### Strengths and limitations

The strengths of our study include its prospective design, large sample size, extended follow-up duration, comprehensive adjustment for potential confounders, and robust sensitivity analyses. The use of a population-based biobank enhances generalizability, and the consistency of findings across multiple analytical approaches strengthens causal inference. Furthermore, our demonstration of effect modification through both continuous dose–response modeling and categorical joint analyses provides complementary evidence for the diet-immune interaction.

Several limitations should be acknowledged. First, as an observational study, residual confounding cannot be fully excluded despite multivariable adjustment. Second, dietary quality was assessed using a baseline Oxford WebQ 24-h recall, and monocyte count was measured at a single baseline time point; these single measurements may not fully capture long-term exposure or inflammatory burden, and resulting non-differential misclassification/regression dilution may have attenuated the observed associations toward the null. Third, we assessed total monocyte counts rather than specific monocyte subsets, limiting mechanistic resolution. Fourth, the proteomic and metabolomic analyses were exploratory and conducted in a subset of participants, so these findings should be interpreted cautiously. Fifth, the final analytic sample was derived through complete-case restriction after sequential exclusions, which may have introduced selection bias and limited generalizability. Finally, the dietary quality index used in this study was a targeted measure rather than a universal dietary quality index. Future studies should examine its concordance with validated inflammatory dietary indices to better assess its construct validity and broader applicability.

### Future research directions

Several avenues for future investigation emerge from our findings. First, randomized controlled trials are needed to establish causality and determine optimal dietary interventions for individuals with elevated inflammatory markers. Such trials should incorporate detailed monocyte subset characterization, gut microbiome profiling, and comprehensive metabolomic assessment to elucidate mechanistic pathways. Second, investigation of other inflammatory cell populations, including neutrophils and lymphocyte subsets, may reveal additional diet-modifiable immune-CVD relationships. Third, integration of multi-omics approaches—combining genomics, transcriptomics, metabolomics, and microbiomics—could identify personalized dietary strategies based on individual immune-metabolic profiles. Finally, implementation science research is needed to develop effective strategies for delivering dietary interventions in clinical practice, particularly for high-risk populations.

## Conclusion

In conclusion, we provide prospective evidence that dietary quality modulates the association. This finding was accompanied by an exploratory proteomic signal implicating REG4, though formal mediation was not established and this result requires replication. Our results support the integration of dietary counseling into cardiovascular risk management, particularly for individuals with evidence of systemic inflammation, and underscore that the inflammatory determinants of CVD are modifiable through lifestyle intervention. Future mechanistic studies and randomized trials are warranted to optimize dietary approaches for immune-cardiovascular health and to translate these findings into precision prevention strategies.

## Data Availability

The data analyzed in this study is subject to the following licenses/restrictions: the data used in this study were accessed through the UK Biobank. The dataset is available to bona fide researchers upon application through the UK Biobank Access Management System (https://www.ukbiobank.ac.uk/). However, access to the UK Biobank data is restricted and requires appropriate approval. The data is subject to conditions of use, and researchers must adhere to the ethical guidelines and access policies set by the UK Biobank. The dataset used in this study is available upon reasonable request, in accordance with UK Biobank’s terms and conditions. Requests to access these datasets should be directed to access the UK Biobank dataset can be made through the UK Biobank Access Management System. The details for application are available on the UK Biobank website: https://www.ukbiobank.ac.uk/. Alternatively, you can contact the UK Biobank directly via email at access@ukbiobank.ac.uk for further information regarding the data access process.
