# Supplementary material for: Healthy diet attenuates the cardiovascular risk associated with elevated monocytes: a prospective cohort study
Source: Front Nutr. 2026 May 26;13:1813578. doi: 10.3389/fnut.2026.1813578 (PMC13246634; doi:10.3389/fnut.2026.1813578)
Supplement: Supplementary file 1 [file Supplementary_file_1.docx]

**Table S1. Sensitivity and subgroup analyses of the interaction between monocyte count and diet quality on incident CVD risk.**

| **Model** | **N** | **Events** | **P_Interaction** |
| --- | --- | --- | --- |
| Main Analysis | 26585 | 1834 | 0.0413 |
| Exclude events < 2 years | 26555 | 1804 | 0.03 |
| Subgroup: Male | 11952 | 1179 | 0.1465 |
| Subgroup: Female | 14633 | 655 | 0.1868 |
| Subgroup: Age >= 60 | 11210 | 1173 | 0.2220 |
| Subgroup: Age < 60 | 15375 | 661 | 0.0410 |
| Alternative Metric: MLR | 26585 | 1834 | 0.0347 |

Values are hazard ratios (HRs) and 95% confidence intervals (CIs) for the multiplicative interaction term (Monocyte Z-score \times Diet Quality Z-score). An HR < 1.00 indicates that the association between monocyte count and CVD risk is attenuated by higher diet quality.

Abbreviations: CVD, cardiovascular disease; HR, hazard ratio; CI, confidence interval; MLR, monocyte count/lymphocyte count ratio.

Main Analysis was adjusted for age, sex, BMI, smoking status, alcohol consumption, and Townsend deprivation index.

Sensitivity Analysis excluded participants who developed CVD within the first 2 years of follow-up to minimize reverse causality.

Subgroup Analyses were adjusted for the same covariates as the main analysis, excluding the stratification variable (e.g., sex was excluded from the model in male/female subgroups).

**Table S2.** **Joint association of peripheral monocyte counts and diet quality with the risk of incident cardiovascular disease. (Numerical results for Figure 3)**

| **Group** | **HR** | **LCI** | **UCI** | ***P*** |
| --- | --- | --- | --- | --- |
| LowMono_HealthyDiet | 1 | 1 | 1 | NA |
| LowMono_UnhealthyDiet | 1.0751350343451749 | 0.9290640611244394 | 1.2441718396440873 | 0.3308567683456563 |
| HighMono_HealthyDiet | 1.0425473547186273 | 0.9086086643201887 | 1.1962300487680446 | 0.5525786261817563 |
| HighMono_UnhealthyDiet | 1.1757712885650489 | 1.027639490740463 | 1.3452559340803487 | 0.018433406992792126 |

Data are presented as Hazard Ratios (HR) and 95% Confidence Intervals (CI). The "Low Monocyte + Healthy Diet" group serves as the reference category (HR = 1.00).

Abbreviations: HR, hazard ratio; LCI, lower confidence interval; UCI, upper confidence interval; NA, not applicable.

Model adjustments: The Cox proportional hazards model was adjusted for age, sex, body mass index, smoking status, alcohol consumption, and socioeconomic status.

**Table S3. Interaction between monocyte count (z score) and diet quality on REG4 protein levels: results from models with progressive adjustment**

| **Model** | **Sample size (N)** | **Beta coefficient** | **Standard error** | **P value for interaction** |
| --- | --- | --- | --- | --- |
| Original Model | 2738 | -0.08188 | 0.02007 | 1.03 × 10⁻⁴ |
| Fully Adjusted Model | 2738 | -0.08444 | 0.02011 | 2.69 × 10⁻⁵ |

Beta coefficient represents the interaction effect for monocyte count z score × high diet quality (reference: low diet quality) on REG4 protein levels.

All models are linear regressions with complete-case analysis.

Original Model: Adjusted for age, sex, and BMI.

Fully Adjusted Model: Additionally adjusted for smoking status, alcohol consumption, socioeconomic status (SES), and log-transformed C-reactive protein (log-CRP z score).

**Table S4. Sensitivity analyses of alternative Diet Quality Index (DQI) constructions for the interaction between standardized monocyte count and incident cardiovascular disease**

| **Analysis** | **N** | **Events** | **HR (95% CI)** | **P-value** |
| --- | --- | --- | --- | --- |
| Original DQI | 26,585 | 1,834 | 0.955 (0.914–0.998) | 0.040 |
| Equal-weight DQI | 26,585 | 1,834 | 0.954 (0.915–0.996) | 0.031 |
| Extended DQI (Recommended) | 26,585 | 1,834 | 0.949 (0.908–0.991) | 0.019 |
| LOO: Excluding Meat | 26,585 | 1,834 | 0.949 (0.909–0.993) | 0.018 |
| LOO: Excluding Fish | 26,585 | 1,834 | 0.949 (0.910–0.993) | 0.018 |
| LOO: Excluding Plant | 26,585 | 1,834 | 0.949 (0.915–0.999) | 0.019 |

Note: HR represents the hazard ratio for the interaction term between standardized monocyte count and standardized DQI score in the Cox proportional hazards model. An HR < 1 indicates that higher diet quality weakens the association between higher monocyte count and incident CVD risk. DQI scores were standardized in each analysis to allow direct comparison across score versions. The extended DQI was the primary version used in the main analyses. LOO denotes leave-one-out sensitivity analyses in which one dietary component was sequentially excluded to assess whether the observed interaction was driven by any single component. All interaction terms were nominally significant (all P < 0.05). CI, confidence interval.

**Table S5. Composition and scoring framework of alternative DQI versions**

| **DQI Version** | **Components Included** | **Total Components** | **Scoring / Weighting Approach** | **Notes** |
| --- | --- | --- | --- | --- |
| Original DQI | Processed/red meat, fish, plant-based foods | 3 | Initial scoring: meat penalty ×2.5, fish ×3, plant ×2; total summed and scaled to 0–100 | Baseline version using only three core components |
| Equal-weight DQI | Processed/red meat, fish, plant-based foods | 3 | All three components assigned equal weight (standardized then averaged and scaled to 0–100) | Used to test sensitivity to differential weighting |
| Extended DQI | Processed/red meat, fish, plant-based foods, sodium, alcohol, whole grains | 6 | Refined coefficients for core components (meat ×2.0, fish ×2.5, plant ×1.8); added sodium (penalty), alcohol (J-shaped scoring: 10/8/5/0 points), whole grains (×2); each component 0–10 points, total /60 ×100 | Primary version; expands the index with three additional inflammation- and CVD-relevant components while refining core weights based on biological plausibility |
| LOO: Excluding meat | Fish, plant-based foods, sodium, alcohol, whole grains | 5 | Same as Extended DQI, with meat removed and score rescaled accordingly | Leave-one-out sensitivity analysis |
| LOO: Excluding fish | Processed/red meat, plant-based foods, sodium, alcohol, whole grains | 5 | Same as Extended DQI, with fish removed and score rescaled accordingly | Leave-one-out sensitivity analysis |
| LOO: Excluding plant-based foods | Processed/red meat, fish, sodium, alcohol, whole grains | 5 | Same as Extended DQI, with plant-based foods removed and score rescaled accordingly | Leave-one-out sensitivity analysis |

Note: All DQI versions were derived from baseline Oxford WebQ 24-hour dietary recall data. Detailed variable definitions and exact scoring algorithms are provided in the Supplementary Methods. In all interaction models, DQI scores were standardized to enable direct comparison of effect sizes across score versions. LOO, leave-one-out.

**Table S6. Tests of proportional hazards assumptions for sequential Cox models of monocyte counts and CVD incidence in the UK Biobank cohort.**

| **Model** | **Term** | **Chi-square** | **Degrees of freedom** | ***P* value** |
| --- | --- | --- | --- | --- |
| Model 1: Crude | Monocyte count (z score) | 7.488 | 1 | 0.006 |
| Model 1: Crude | GLOBAL | 7.488 | 1 | 0.006 |
| Model 2: Age + Sex | Monocyte count (z score) | 7.889 | 1 | 0.005 |
| Model 2: Age + Sex | Age | 0.907 | 1 | 0.341 |
| Model 2: Age + Sex | Sex | 3.652 | 1 | 0.056 |
| Model 2: Age + Sex | GLOBAL | 10.119 | 3 | 0.018 |
| Model 3: Main adjusted | Monocyte count (z score) | 7.660 | 1 | 0.006 |
| Model 3: Main adjusted | Age | 0.884 | 1 | 0.347 |
| Model 3: Main adjusted | Sex | 3.612 | 1 | 0.057 |
| Model 3: Main adjusted | BMI | 1.022 | 1 | 0.312 |
| Model 3: Main adjusted | Smoking category | 1.465 | 2 | 0.481 |
| Model 3: Main adjusted | Alcohol category | 0.166 | 2 | 0.920 |
| Model 3: Main adjusted | GLOBAL | 13.453 | 8 | 0.097 |
| Model 4: Main adjusted + SES | Monocyte count (z score) | 7.653 | 1 | 0.006 |
| Model 4: Main adjusted + SES | Age | 0.875 | 1 | 0.349 |
| Model 4: Main adjusted + SES | Sex | 3.601 | 1 | 0.058 |
| Model 4: Main adjusted + SES | BMI | 0.984 | 1 | 0.321 |
| Model 4: Main adjusted + SES | Smoking category | 1.463 | 2 | 0.481 |
| Model 4: Main adjusted + SES | Alcohol category | 0.160 | 2 | 0.923 |
| Model 4: Main adjusted + SES | SES | 0.297 | 1 | 0.586 |
| Model 4: Main adjusted + SES | GLOBAL | 13.547 | 9 | 0.139 |
| Model 5: Main adjusted + SES + CRP | Monocyte count (z score) | 7.558 | 1 | 0.006 |
| Model 5: Main adjusted + SES + CRP | Age | 0.870 | 1 | 0.351 |
| Model 5: Main adjusted + SES + CRP | Sex | 3.599 | 1 | 0.058 |
| Model 5: Main adjusted + SES + CRP | BMI | 1.002 | 1 | 0.317 |
| Model 5: Main adjusted + SES + CRP | Smoking category | 1.481 | 2 | 0.477 |
| Model 5: Main adjusted + SES + CRP | Alcohol category | 0.160 | 2 | 0.923 |
| Model 5: Main adjusted + SES + CRP | SES | 0.300 | 1 | 0.584 |
| Model 5: Main adjusted + SES + CRP | Log CRP (z score) | 0.801 | 1 | 0.371 |
| Model 5: Main adjusted + SES + CRP | GLOBAL | 16.195 | 10 | 0.094 |

Note: P values were derived from Schoenfeld residual tests. The Schoenfeld residual test for monocyte count was statistically significant across all sequential models, suggesting potential non-proportionality for this covariate. However, the global Schoenfeld tests were not statistically significant in the more fully adjusted models (Models 3–5), indicating no strong evidence of overall violation of the proportional hazards assumption at the model level in the primary analyses.

**Figure S1. Time-varying association between standardized monocyte count and incident cardiovascular disease**


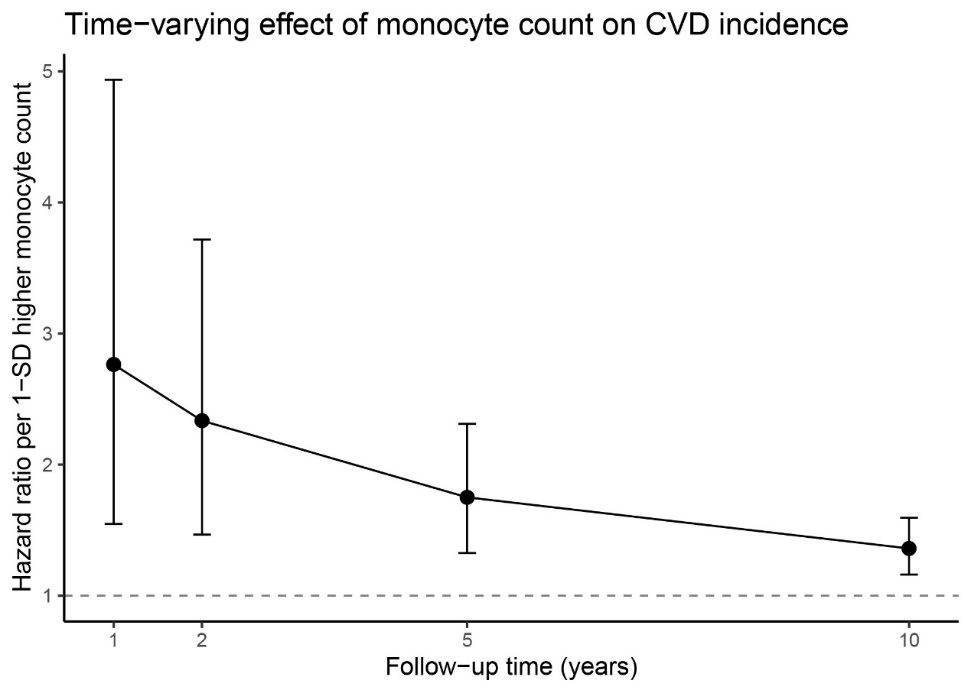


Estimated hazard ratios for incident cardiovascular disease per 1-SD higher standardized monocyte count are shown at 1, 2, 5, and 10 years of follow-up. The association was strongest during early follow-up and weakened over time, consistent with a modest departure from the proportional hazards assumption. Error bars represent 95% confidence intervals.

**Table S7. Variance inflation factor diagnostics for sequential Cox models of monocyte counts and CVD incidence in the UK Biobank cohort.**

| **Model** | **Variable** | **VIF** |
| --- | --- | --- |
| Model 2: Age + Sex | Monocyte count (z score) | 1.06 |
| Model 2: Age + Sex | Age | 1.01 |
| Model 2: Age + Sex | Sex | 1.05 |
| Model 3: Main adjusted | Monocyte count (z score) | 1.09 |
| Model 3: Main adjusted | Age | 1.03 |
| Model 3: Main adjusted | Sex | 1.08 |
| Model 3: Main adjusted | BMI | 1.04 |
| Model 3: Main adjusted | Smoking (previous) | 1.08 |
| Model 3: Main adjusted | Smoking (current) | 1.06 |
| Model 3: Main adjusted | Alcohol (occasional) | 2.50 |
| Model 3: Main adjusted | Alcohol (regular) | 2.51 |
| Model 4: Main adjusted + SES | Monocyte count (z score) | 1.09 |
| Model 4: Main adjusted + SES | Age | 1.04 |
| Model 4: Main adjusted + SES | Sex | 1.08 |
| Model 4: Main adjusted + SES | BMI | 1.05 |
| Model 4: Main adjusted + SES | Smoking (previous) | 1.08 |
| Model 4: Main adjusted + SES | Smoking (current) | 1.08 |
| Model 4: Main adjusted + SES | Alcohol (occasional) | 2.50 |
| Model 4: Main adjusted + SES | Alcohol (regular) | 2.52 |
| Model 4: Main adjusted + SES | SES | 1.03 |
| Model 5: Main adjusted + SES + CRP | Monocyte count (z score) | 1.11 |
| Model 5: Main adjusted + SES + CRP | Age | 1.05 |
| Model 5: Main adjusted + SES + CRP | Sex | 1.09 |
| Model 5: Main adjusted + SES + CRP | BMI | 1.28 |
| Model 5: Main adjusted + SES + CRP | Smoking (previous) | 1.08 |
| Model 5: Main adjusted + SES + CRP | Smoking (current) | 1.09 |
| Model 5: Main adjusted + SES + CRP | Alcohol (occasional) | 2.50 |
| Model 5: Main adjusted + SES + CRP | Alcohol (regular) | 2.52 |
| Model 5: Main adjusted + SES + CRP | SES | 1.03 |
| Model 5: Main adjusted + SES + CRP | Log CRP (z score) | 1.30 |

Abbreviations: BMI, body mass index; CRP, C-reactive protein; SES, socioeconomic status; VIF, variance inflation factor.

Note: All VIF values were low, with the highest value below 3, indicating no evidence of problematic multicollinearity among monocyte count, BMI, CRP, and other covariates included in the sequential Cox models.

**Table S8. Association between monocyte count and CVD incidence in UKB cohort (cause-specific Cox).**

| **Peripheral immune markers** |  | **Crude model (HR, 95% CI)** | ***P* value** | ***P* for trend** | **CRP-adjusted sensitivity model (HR, 95% CI)** | ***P* value** | ***P* for trend** |
| --- | --- | --- | --- | --- | --- | --- | --- |
| **Monocyte Count** | Continuous | 1.56 (1.36-1.79) | < 0.0001 | < 0.0001 | 1.25 (1.08-1.45) | 0.0030 | 0.0203 |
|  | Q1 | 1.00 (Reference) |  |  | 1.00 (Reference) |  |  |
|  | Q2 | 1.05 (0.60-1.83) | 0.8743 |  | 0.87 (0.49-1.52) | 0.6162 |  |
|  | Q3 | 1.35 (0.80-2.29) | 0.2656 |  | 0.98 (0.57-1.67) | 0.9415 |  |
|  | Q4 | 2.85 (1.78-4.54) | < 0.0001 |  | 1.51 (0.93-2.46) | 0.0974 |  |

Abbreviations: HR, hazard ratio; CI, confidence interval; Ref, reference; CRP, C-reactive protein.

Crude model: unadjusted.

CRP-adjusted sensitivity model: adjusted for age, sex, body mass index, smoking status, alcohol consumption, socioeconomic status, and CRP.

P for trend: quartile-specific median monocyte values were entered as a continuous term in the Cox model.

In the cause-specific Cox model, participants experiencing competing non-CVD death were censored at the time of death.

Analysis sample size: N = 26,585; number of incident CVD events: 1,834.

**Table S9. Detailed association of monocyte levels with incident cardiovascular disease across sequential Cox proportional hazards models**

**A.** Association between monocyte quartiles and incident cardiovascular disease across sequential Cox proportional hazards models

| **Model** | **Exposure** | **N** | **Person-years** | **Events, n (%)** | **HR (95% CI)** | **P value** |
| --- | --- | --- | --- | --- | --- | --- |
| Model 1 | Q1 (Ref) | 6,646 | 108,931.2 | 296 (4.5%) | 1.00 (Ref) |  |
|  | Q2 vs Q1 | 6,646 | 108,677.4 | 311 (4.7%) | 0.92 (0.53-1.62) | 0.7773 |
|  | Q3 vs Q1 | 6,646 | 108,301.7 | 398 (6.0%) | 1.08 (0.64-1.85) | 0.7688 |
|  | Q4 vs Q1 | 6,647 | 106,962.0 | 829 (12.5%) | 1.94 (1.20-3.13) | 0.0066 |
| Model 2 | Q1 (Ref) | 6,646 | 108,931.2 | 296 (4.5%) | 1.00 (Ref) |  |
|  | Q2 vs Q1 | 6,646 | 108,677.4 | 311 (4.7%) | 0.88 (0.50-1.54) | 0.6464 |
|  | Q3 vs Q1 | 6,646 | 108,301.7 | 398 (6.0%) | 0.99 (0.58-1.69) | 0.9759 |
|  | Q4 vs Q1 | 6,647 | 106,962.0 | 829 (12.5%) | 1.61 (0.99-2.61) | 0.0553 |
| Model 3 | Q1 (Ref) | 6,646 | 108,931.2 | 296 (4.5%) | 1.00 (Ref) |  |
|  | Q2 vs Q1 | 6,646 | 108,677.4 | 311 (4.7%) | 0.87 (0.50-1.53) | 0.6352 |
|  | Q3 vs Q1 | 6,646 | 108,301.7 | 398 (6.0%) | 1.00 (0.59-1.70) | 0.9965 |
|  | Q4 vs Q1 | 6,647 | 106,962.0 | 829 (12.5%) | 1.59 (0.98-2.58) | 0.0603 |

**B.** Association between continuous monocyte level and incident cardiovascular disease across sequential Cox proportional hazards models

| **Model** | **Exposure** | **N** | **Person-years** | **Events, n (%)** | **HR (95% CI)** | **P value** |
| --- | --- | --- | --- | --- | --- | --- |
| Model 1 | Continuous monocyte (per SD) | 26,585 | 432,872.4 | 1834 (6.9%) | 1.38 (1.20-1.59) | <0.0001 |
| Model 2 | Continuous monocyte (per SD) | 26,585 | 432,872.4 | 1834 (6.9%) | 1.29 (1.11-1.49) | 0.0007 |
| Model 3 | Continuous monocyte (per SD) | 26,585 | 432,872.4 | 1834 (6.9%) | 1.28 (1.11-1.48) | 0.0009 |

**C.** Test for linear trend across monocyte quartiles

| **Model** | **Trend term** | **Beta** | **SE** | **P for trend** |
| --- | --- | --- | --- | --- |
| Model 1 | Median value of monocyte quartiles | 2.387 | 0.682 | 0.0005 |
| Model 2 | Median value of monocyte quartiles | 1.815 | 0.691 | 0.0087 |
| Model 3 | Median value of monocyte quartiles | 1.785 | 0.690 | 0.0098 |

Abbreviations: HR, hazard ratio; CI, confidence interval; Ref, reference.

Model 1 was adjusted for age and sex.

Model 2 was additionally adjusted for body mass index, smoking status, and alcohol consumption.

Model 3 was additionally adjusted for socioeconomic status.

P for trend was calculated by assigning the median monocyte value within each quartile to all participants in that quartile and entering this ordered variable as a continuous term in the Cox proportional hazards model.

In the cause-specific Cox model, participants who died from causes other than cardiovascular disease were censored at the time of death.

The continuous and quartile analyses were conducted in the same analytic sample (N = 26,585; total person-years = 432,872.4; incident CVD events = 1,834).

This supplementary table provides the detailed stratum-specific participant counts, person-years, event counts, and trend statistics corresponding to the summary results presented in Table 2 of the main text.

A CRP-adjusted sensitivity model, additionally adjusted for log-CRP, is reported in ***Supplementary Table 8***.

**Table S10. Top 20 proteins from the proteome-wide interaction screening analysis**

| **Protein ID** | **Protein Name** | **Interaction *P* value** | **Interaction beta** | **FDR *q* value** |
| --- | --- | --- | --- | --- |
| 2267 | REG4 (Regenerating islet-derived protein 4) | 2.69435E-05 | -0.084432673 | 0.078755716 |
| 1430 | INHBB (Inhibin beta B chain) | 0.00017941 | -0.078011244 | 0.219952174 |
| 2686 | TIMP2 (Metalloproteinase inhibitor 2) | 0.000225746 | -0.036618186 | 0.219952174 |
| 2254 | RBP2 (Retinol-binding protein 2) | 0.000608418 | -0.112564932 | 0.444601115 |
| 2607 | SWAP70 (Switch-associated protein 70) | 0.000760931 | -0.04725119 | 0.444840379 |
| 509 | CEACAM20 (Carcinoembryonic antigen-related cell adhesion molecule 20) | 0.001049123 | -0.057243683 | 0.476732114 |
| 2235 | RAD51 (DNA repair protein RAD51 homolog 1) | 0.001141678 | -0.076413727 | 0.476732114 |
| 462 | CD70 (CD70 antigen) | 0.001316682 | -0.060655744 | 0.481082854 |
| 51 | ADGRD1 (Adhesion G-protein coupled receptor D1) | 0.001775176 | -0.049546575 | 0.524480453 |
| 994 | FABP6 (Gastrotropin) | 0.001794322 | -0.070466327 | 0.524480453 |
| 2156 | PRRT3 (Proline-rich transmembrane protein 3) | 0.002053137 | -0.047626358 | 0.5386238 |
| 1037 | FGF2 (Fibroblast growth factor 2) | 0.002459146 | -0.091375834 | 0.5386238 |
| 118 | ANGPT2 (Angiopoietin-2) | 0.002631418 | -0.048815189 | 0.5386238 |
| 2216 | QDPR (Dihydropteridine reductase) | 0.002774029 | -0.054774028 | 0.5386238 |
| 1648 | LYVE1 (Lymphatic vessel endothelial hyaluronic acid receptor 1) | 0.0028582 | -0.024484407 | 0.5386238 |
| 1999 | PDCD6 (Programmed cell death protein 6) | 0.002974202 | -0.087238032 | 0.5386238 |
| 1158 | GHRL (Appetite-regulating hormone) | 0.003754105 | -0.099028064 | 0.5386238 |
| 2263 | REG1A (Lithostathine-1-alpha) | 0.00393227 | -0.059156842 | 0.5386238 |
| 2264 | REG1B (Lithostathine-1-beta) | 0.004783784 | -0.068068952 | 0.5386238 |
| 1398 | IL22 (Interleukin-22) | 0.005343441 | -0.076388736 | 0.5386238 |

Abbreviations: FDR, false discovery rate.

Note: Proteins are ranked by nominal P value for the interaction between monocyte count and diet quality. FDR q values were calculated using the Benjamini–Hochberg procedure across all 2,924 proteins tested. No protein met the prespecified FDR significance threshold of q < 0.05. REG4 showed the smallest nominal interaction P value and the lowest FDR q value, but did not meet the FDR threshold. Accordingly, the proteins listed here should be interpreted as exploratory interaction-associated signals rather than statistically robust findings after multiple-testing correction.

**Table S11. Participant selection for the monocyte-CVD analysis and proteomic subsample**

| **Order** | **Selection step** | **N** | **Excluded at this step** | **Criterion** |
| --- | --- | --- | --- | --- |
| 1 | Initial study sample | 502,356 | 0 | Starting dataset |
| 2 | Participants with complete dietary variables | 70,679 | 431,677 | Missing dietary variables |
| 3 | Participants within plausible energy intake range | 69,521 | 1,158 | Energy intake outside predefined range |
| 4 | Participants within protein energy percentage range | 69,457 | 64 | Protein energy percentage outside predefined range |
| 5 | Participants with complete baseline covariates | 41,391 | 28,066 | Missing baseline covariates |
| 6 | Final analytic sample for monocyte-CVD analysis | 26,585 | 14,806 | Missing follow-up, outcome, monocyte count, or key covariates |
| 7 | Proteomic subsample | 2,738 | 23,847 | Availability of proteomic data |

Note: The final analytic sample was derived through sequential exclusions based on dietary data completeness, plausibility filters, availability of baseline covariates, and complete-case requirements for the monocyte-CVD analysis. The proteomic subsample was nested within the final analytic sample and included participants with available proteomic measurements.

**Table S12. Fine-Gray competing-risk regression analysis of monocyte count and incident CVD, with non-CVD death as the competing event**

| **Peripheral immune markers** | **Group** | **M1 sHR (95% CI)** | ***P* value** | ***P* for trend** | **M2 sHR (95% CI)** | ***P* value** | ***P* for trend** | **M3 sHR (95% CI)** | ***P* value** | ***P* for trend** | **M4 sHR (95% CI)** | ***P* value** | ***P* for trend** | **M5 sHR (95% CI)** | ***P* value** | ***P* for trend** |
| --- | --- | --- | --- | --- | --- | --- | --- | --- | --- | --- | --- | --- | --- | --- | --- | --- |
| Monocyte count | Continuous (per 1 SD) | 1.54 (1.34-1.77) | < 0.0001 | < 0.0001 | 1.37 (1.18-1.60) | < 0.0001 | 0.0011 | 1.28 (1.10-1.50) | 0.0019 | 0.0142 | 1.28 (1.09-1.49) | 0.0022 | 0.015 | 1.25 (1.06-1.46) | 0.0065 | 0.0294 |
|  | Q1 | 1.00 (Reference) |  |  | 1.00 (Reference) |  |  | 1.00 (Reference) |  |  | 1.00 (Reference) |  |  | 1.00 (Reference) |  |  |
|  | Q2 | 1.04 (0.60-1.83) | 0.8838 |  | 0.92 (0.52-1.63) | 0.7841 |  | 0.88 (0.50-1.55) | 0.6619 |  | 0.88 (0.50-1.55) | 0.6569 |  | 0.87 (0.49-1.54) | 0.6354 |  |
|  | Q3 | 1.33 (0.79-2.26) | 0.2857 |  | 1.08 (0.63-1.83) | 0.7853 |  | 0.99 (0.58-1.69) | 0.9822 |  | 1.00 (0.59-1.70) | 0.9996 |  | 0.98 (0.58-1.67) | 0.9466 |  |
|  | Q4 | 2.76 (1.73-4.40) | < 0.0001 |  | 1.92 (1.18-3.11) | 0.0086 |  | 1.60 (0.97-2.62) | 0.0635 |  | 1.59 (0.97-2.60) | 0.0669 |  | 1.51 (0.91-2.49) | 0.1074 |  |

Abbreviations: sHR, subdistribution hazard ratio; CI, confidence interval; Ref, reference; CRP, C-reactive protein; SES, socioeconomic status.

Note: Fine-Gray subdistribution hazard models were used, with non-CVD death treated as the competing event. Model 1 was unadjusted. Model 2 was adjusted for age and sex. Model 3 was additionally adjusted for BMI, smoking status, and alcohol consumption. Model 4 was further adjusted for SES. Model 5 was further adjusted for log-transformed CRP. The direction and overall pattern of association were consistent with those observed in the primary cause-specific Cox models, with attenuation of effect estimates after multivariable adjustment.

**Table S13. Exploratory analysis of potential metabolic mediators.**

| **Variable** | **N** | **Beta (SE)** | **P Interaction** |
| --- | --- | --- | --- |
| **Glycemic** **Traits** |  |  |  |
| Fasting Glucose | 26585 | -0.016 (0.006) | 0.104 |
| **Anthropometry** |  |  |  |
| Hip Circumference | 26582 | 0.043 (0.028) | 0.1259 |
| **Renal Function** |  |  |  |
| Serum Creatinine | 26585 | 0.001 (0.001) | 0.3076 |
| eGFR | 26585 | -0.057 (0.067) | 0.3994 |
| **Inflammation** |  |  |  |
| C-Reactive Protein | 26585 | -0.005 (0.006) | 0.3814 |
| **Lipids & Fatty Acids** |  |  |  |
| LA (Linoleic Acid) | 14675 | 0.005 (0.006) | 0.3505 |
| SFA (Saturated Fatty Acids) | 14675 | -0.007 (0.007) | 0.3643 |
| MUFA (Monounsaturated Fatty Acids) | 14675 | -0.005 (0.006) | 0.3943 |
| SM (Sphingomyelins) | 14675 | -0.000 (0.001) | 0.3955 |
| Omega-3 | 14675 | -0.003 (0.002) | 0.1206 |
| TFA (Total Fatty Acids) | 14675 | -0.013 (0.019) | 0.4986 |
| HDL-C (High-Density Lipoprotein Cholesterol) | 14688 | -0.001 (0.002) | 0.5175 |
| DHA (Docosahexaenoic Acid) | 14675 | -0.000 (0.001) | 0.5495 |
| TEC (Total Esterified Cholesterol) | 14688 | -0.002 (0.005) | 0.6467 |
| TC (Total Cholesterol) | 14688 | -0.003 (0.007) | 0.6844 |
| Omega-6 | 14675 | 0.002 (0.005) | 0.7168 |
| PC (Phosphatidylcholines) | 14675 | 0.001 (0.003) | 0.7393 |
| TFC (Total Free Cholesterol) | 14688 | -0.001 (0.002) | 0.7867 |
| LDL-C (Low-Density Lipoprotein Cholesterol) | 14688 | -0.001 (0.004) | 0.8024 |
| TLPP (Total Lipoprotein Particles) | 14688 | -0.000 (0.000) | 0.8082 |
| ApoA1 (Apolipoprotein A1) | 14688 | -0.000 (0.002) | 0.8306 |
| ApoB (Apolipoprotein B) | 14688 | -0.000 (0.002) | 0.8394 |
| PG (Phosphoglycerides) | 14675 | -0.000 (0.003) | 0.8721 |
| TG (Triglycerides) | 14688 | -0.001 (0.004) | 0.8851 |
| ApoB/ApoA1 Ratio | 14688 | 0.000 (0.001) | 0.8865 |
| VLDL-C (Very-Low-Density Lipoprotein Cholesterol) | 14688 | 0.000 (0.002) | 0.8941 |
| PUFA (Polyunsaturated Fatty Acids) | 14675 | -0.001 (0.006) | 0.9113 |
| TCHOL (Total Cholines) | 14675 | -0.000 (0.003) | 0.9322 |

Values are beta coefficients (standard errors) representing the change in the slope of the association between monocyte count (Z-score) and each biomarker per unit increase in diet quality, derived from linear regression models.

Abbreviations: SE, standard error; FDR, false discovery rate; eGFR, estimated glomerular filtration rate; LA, linoleic acid; SFA, saturated fatty acids; MUFA, monounsaturated fatty acids; PUFA, polyunsaturated fatty acids; TFA, total fatty acids; DHA, docosahexaenoic acid; SM, sphingomyelins; PC, phosphatidylcholines; PG, phosphoglycerides; TC, total cholesterol; LDL-C, low-density lipoprotein cholesterol; HDL-C, high-density lipoprotein cholesterol; VLDL-C, very-low-density lipoprotein cholesterol; TG, triglycerides; ApoA1, apolipoprotein A1; ApoB, apolipoprotein B.

Models were adjusted for age, sex, body mass index, smoking status, alcohol consumption, and socioeconomic status. *P* for Interaction tests whether the association between monocyte count and the biomarker differs by diet quality.
